# Cutaneous Leishmaniasis in an Immigrant Saudi Worker: A Case Report

**Published:** 2014-06

**Authors:** Hafizur Rahman, Mohammad A. Razzak, Bikash C. Chanda, Khondaker R.H. Bhaskar, Dinesh Mondal

**Affiliations:** ^1^Clinical Laboratory Services, Diagnostic Labs, Laboratories, icddr,b, GPO Box 128, Dhaka 1000, Bangladesh; ^2^Centre for Nutrition and Food Security, Parasitology Laboratory, Laboratories, icddr,b, GPO Box 128, Dhaka 1000, Bangladesh

**Keywords:** Cutaneous leishmaniasis, LD bodies, *Leishmania tropica*, Sodium stibogluconate, Bangladesh

## Abstract

Cutaneous leishmaniasis (CL), an uncommon disorder in South-East Asia, including Bangladesh, often presents as granulomatous plaque on the exposed areas, with a high index of suspicion required for diagnosis. Here we report the first imported case of CL caused by *Leishmania tropica* in a migrant Bangladeshi worker in the Kingdom of Saudi Arabia (KSA). The case, initially suspected as a case of cutaneous tuberculosis, arrived at specimens reception unit (SRU) of diagnostic labs of icddr,b being referred by the physician for ALS testing for tuberculosis. At his arrival in the SRU, one of the health personnel of the unit who used to work in KSA suspected him as a case of CL. The diagnosis was confirmed by smear microscopy which revealed plenty of amastigotes within macrophages. PCR was performed to confirm the species. He was treated with sodium stibogluconate at Shahid Suhrawardy Medical College Hospital, Dhaka.

## INTRODUCTION

Tropical infections caused by *Leishmania* spp. can present diagnostic problems both to physician as well as the dermatologist. The clinical diagnosis is not difficult with typical features of leishmaniasis in endemic countries. However, in non-endemic countries where cutaneous leishmaniasis (CL) is not common as in Bangladesh, it can easily be missed. When considering a cutaneous lesion of possible infective cause, the common differentials would include mycobacterial and deep fungal infections. In a country like ours where tuberculosis is more prevalent, cutaneous leishmaniasis is very likely to be mistreated as cutaneous tuberculosis, especially lupus vulgaris. Here we report a case of cutaneous leishmaniasis who presented to the specimens reception unit of diagnostic lab of icddr,b in December 2011 and was probably imported from Kingdom of Saudi Arabia where the patient used to work in the past.

## CASE REPORT

**Figure 1.**
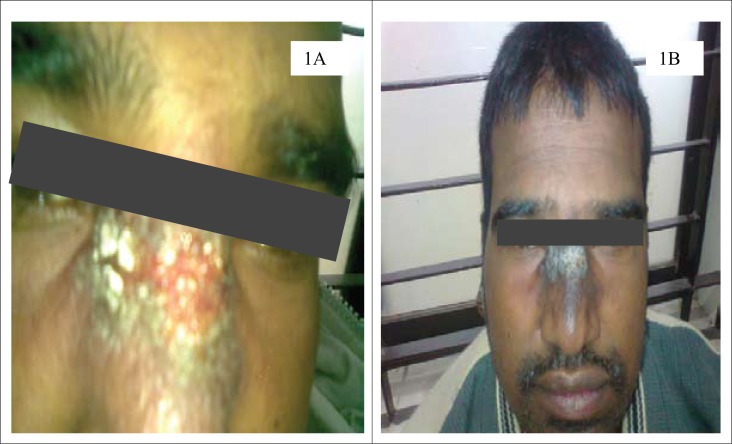
(A) Lesion before treatmentand; (B) Lesion healed with scarring after 10 days of treatment

**Figure 2.**
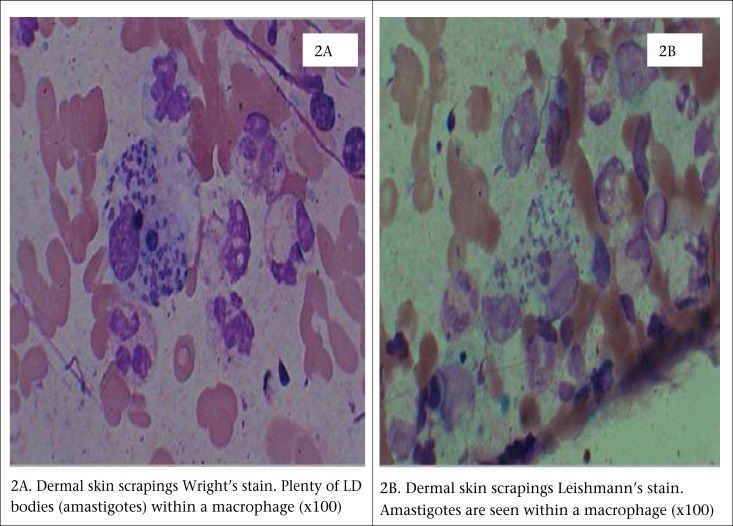
Dermal skin scrapings: (2A) Wright's and (2B) Leishmann's stains

**Figure 3.**
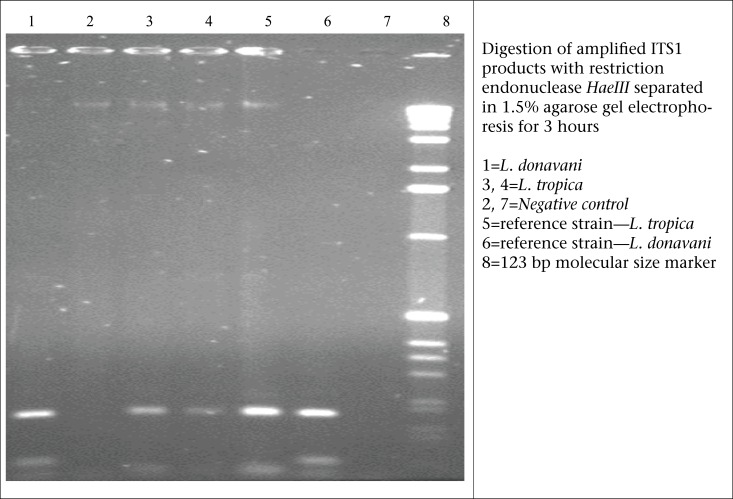
PCR of amplified ITS1 products for detection of *L. donovani* and *L. tropica*

A previously well, 37-year old young adult presented to the SRU of diagnostic labs of icddr,b, with a 6-week history of skin lesions on his nose, ear, arm, and fingers. He was referred to the SRU of diagnostic labs for ALS testing for tuberculosis, along with culture for pus from wound. However, the health personnel in the SRU suspected the case as a possible case of CL based on his experience when he used to work in KSA. Then, the patient was referred to one of the experts of icddr,b in this field. Physical examination revealed painless erythematous papules and nodules with overlying scale and crust, some of which had central ulceration (Figure 1A and 1B) [The patient provided his written consent to the authors to use his photograph in this case study]. The patient was from Saudi Arabia, an area in which cutaneous leishmaniasis is endemic. He remembered being bitten by sandflies during his stay at Saudi Arabia. Following the bite, the lesion started as an itchy red papule slowly enlarged into an inflammatory papule to an ulcer. The lesions usually appear in non-covered regions of the body, mainly the face, nose, ear lobules, elbows, and fingers. The incubation period could not be confirmed from history; it was between 3 and 4 weeks. Systemic examination was unremarkable. The ulcer failed to heal, despite several course of systemic antibiotics. There was no past medical or drug history of note. Based on history and clinical examination, a provisional diagnosis of CL was made. Thin smear from dermal scrapings revealed large macrophage containing abundant intracellular *Leishman-Donovan* bodies (amastigotes); some were free-lying in the dermis without any granuloma (Figure 2); tissue culture and polymerase chain reaction (PCR) confirmed infecting agent as *Leishmania tropica* (Figure 3). He was admitted to Sir Salimullah Medical College Hospital (SSMCH) and treated with sodium stibogluconate (SSG). Intravenous SSG was given at a dose of 20 mg/kg/day for 28 days, along with intralesional injection at a dose of 30 mg/day/lesion for 10 days. A significant improvement was observed after 10 days, and all the ulcers were healed. After 10 days, the patient received only intravenous SSG for another 18 days.

## DISCUSSION

Leishmaniasis is a poverty-related disease caused by several species of the genus *Leishmania*. It affects the poorest of the poor and is associated with malnutrition, displacement, poor housing, illiteracy, gender discrimination, weakness of the immune system, and lack of resources. Leishmaniasis is also linked to environmental changes, such as deforestation, building of dams, new irrigation schemes and urbanization, and the accompanying migration of non-immune people to endemic areas. Each species tends to occupy a particular zoo-geographical zone (1). The clinical manifestations of leishmaniasis depend on the interaction between the characteristic virulence of the species and the host's immune response (2). These are transmitted by the bites of female sandflies of the genus *Phlebotomonas* in the Old World and *Lutzomyia* in the New World. More than 20 species of *Leishmania*, pathogenic for humans and other mammals, have been identified worldwide (3). About 30 species of sandflies are proven vectors; the usual reservoir hosts include humans and domestic/wild animals. The definitive diagnosis depends on demonstration of the parasites by smears, culture, PCR, and histological examination of suspected specimens.

The geographical distribution of leishmaniasis is extremely wide; it is prevalent on our four continents and considered to be endemic in 88 countries, 67 of these being in the Old World and 21 in the New World, including Bangladesh, Brazil, Afghanistan, Iran, Saudi Arabia, Peru, Sudan, and India (4,5). Leishmaniasis in the Old World is endemic in the Mediterranean Sea and the neighbouring countries. The annual incidence worldwide is about 400,000 cases, with a prevalence of approximately 350 million people infected (6). More than 90% of cutaneous leishmaniasis worldwide can be found in Afghanistan, Iran, Saudi Arabia, Syria, Brazil, and Peru. Majority of the cases of cutaneous leishmaniasis are found in adult men between 20 and 40 years (6). Tourists and workers from endemic areas have an increased incidence of CL. CL is mostly imported to endemic countries by immigrants and returning travellers.

Human leishmaniasis is usually classified as visceral, mucosal, or cutaneous. The different forms of the disease are distinct in their causes, epidemiological features, transmission, and geographical distribution. Visceral leishmaniasis (VL) or kala-azar is caused by *L. donovani, L. infantum*, and *L. chagasi*. These species, in contrast with the other species of *Leishmania* that infect man, are normally viscerotropic and cause a severe systemic infection, often accompanied with gross splenomegaly, anaemia, diarrhoea, hepatomegaly, lymphadenopathy, and signs of malnutrition (7). However, a certain percentage of VL may present as post kala-azar dermal leishmianiasis (PKDL) generally after 2-3 years following the treatment for VL, which appears to completely remit. This PKDL also causes a diagnostic dilemma in endemic countries. Mucosal leishmaniasis (ML) is an uncommon but serious manifestation of *Leishmania* infection, resulting from haematogenous metastases to the nasal or oropharyngeal mucosa from a cutaneous infection. It is usually caused by parasites in the *L. (Vianna)* complex. Approximately half of the patients with mucosal lesions have had active cutaneous lesions within the preceding 2 years but ML may not develop until many years after resolution of the primary lesion. ML occurs in <5% of individuals who have or had localized cutaneous leishmaniasis caused by *L. (V.) braziliensis*. Cutaneous leishmaniasis (CL) is mainly caused by *L. tropica, L. major, and L. aetiopica* (8). CL is also known as ‘Aleppo boil’, ‘Baghdad boil’, ‘Bay sore’, ‘Biskra button’, ‘Chiclero ulcer’, ‘Delhi boil’, ‘Kandahar sore’, ‘Lahore sore’, ‘Leishmaniasis tropica’, ‘Oriental sore’, ‘Pian bois’, and ‘Uta’ in respective areas (9). The incubation period in CL is usually measured in months but ranges from a few days to over a year. In our patient, the lesion appeared 3 to 4 weeks after bite of sandflies. He was treated with intravenous and intralesional sodium stibgluconate (850 mg daily) for 28 days to which he responded well. The differential diagnosis is extensive and includes infective granulomas, such as lupus vulgairs, deep fungal infections, mycobacterium infections, leprosy, sarcoidosis, and squamous cell carcinoma. A high index of suspicion is required for provisional diagnosis (10). Many leishmania species and subspecies of the *Leishmania* protozoa have different virulence and clinical predilections; so, treatment should be tailored for every individual. Old World disease tends to be self-limiting. Leishmaiasis caused by this species does not necessarily need to be treated unless the lesion is in a cosmetically- or functionally-sensitive site. In the New World, leishmaniasis treatment is very often the standard of care because of high recurrence rate of chronic ulcers, recidivant lesions, or mucocutaneous involvement.

Treatment of CL is often difficult. Multiple treatment options are used throughout the world for cutaneous disease. Besides oral and parenteral medications (pentavalent antimonials, liposomal amphotericin B, miltefosine, and some others), local cryotherapy, intralesional infiltration of sodium stibogluconate, local heat therapy, and various topical paromomycin preparations are in practice for many many years.

Antimonials are still the first-line drug in the treatment of CL. Sodium stibogluconate (Pentostam) and meglumine antimonite glucantime are essentially similar drugs which contain pentavalent antimony (Sb). Sodium stibogluconate can be administered intravenously or intramuscularly while meglumine antimonite should only be given via the intramuscular route. The recommended dose is 20 mg/kg/day for 20-28 days (8). Treatment with antimonials is associated with some side-effects, such as myalgia as well as possible liver or cardiovascular toxicity, which fortunately is rare. A recent study using intralesional sodium stibogluconate showed that alternate daily or weekly administration of intralesional sodium stibogluconate was effective in the treatment of CL (10). Dapsone and allopurinol have also been used for the treatment of CL. The mechanism is unclear, although basic biomedical studies have shown that *Leishmania* cannot make all of their own nucleic acids and, thus, it uses the host's purine through the purine salvage pathway (1).

Besides systemic treatment, local measures, such as cryotherapy, local excision of a small focus and topical treatment using 15% paromomycin ointment, have also been shown to be effective in some cases (1,11). Vaccines for prophylaxis and immunotherapy have been developed and are currently undergoing trials in many countries, including Venezuela, Brazil, and Iran (1,10). The development of molecular biology techniques is also improving knowledge on the structure, evolution, and expression of the *Leishmania* genome, and the study and definition of the mechanisms that regulate the parasite's biochemical and molecular features will certainly contribute to the development of new and more effective strategies for leishmaniasis treatment. So far, chemotherapy with systemic and intralesional sodium stibogluconate is effective without any major side-effects as was seen in this case.

Visceral leishmaniasis and its skin complication (PKDL) among those treated for VL are common in Bangladesh. Fortunately, there is no report of CL in Bangladesh. This could be due to the absence of vector *P. papattasi* which might be the natural selection and native CL patients (12). However, this should be recalled that there was no reported case of infected vector of dengue fever before 2000 when the first outbreak of dengue fever took place in Bangladesh. Thus, the disease control authority of Bangladesh should be alert about the imported cases of CL. This case report indicates that, if health workers in the immigration are experienced or trained in finding of CL, they can easily be identified at arrival and referred for diagnosis and treatment. This will reduce suffering of such patients; and also lower the risk of disease transmission in community through early case detection and proper management.

### Conclusions

Cutaneous leishmaniasis is a rare disease in Bangladesh. Because of higher rate of travel and work abroad, increased number of sporadic cases of cutaneous leishmaniasis in non-endemic areas should be taken into account. The case emphasizes the point that, when assessing lesions of possible infective aetiology, a detailed travel history and knowledge of the common infective agents in the location concerned are of great importance in arriving at a correct diagnosis for appropriate treatment.

## ACKNOWLEDGEMENTS

Authors gratefully acknowledge icddr,b and all of its core donors which provide unrestricted support to icddr,b for its operations and research. Current donors providing unrestricted support include: Australian Agency for International Development (AusAID); Government of the People's Republic of Bangladesh; Canadian International Development Agency (CIDA); Swedish International Development Cooperation Agency (Sida); and the Department for International Development (DFID), UK.
